# Potential benefits and human systems integration of parastronauts with bilateral vestibulopathy for a space mission

**DOI:** 10.3389/fneur.2025.1556553

**Published:** 2025-03-05

**Authors:** Constance F. Ramsburg, Scott J. Wood, James R. Lackner, Shannan Moynihan, Millard F. Reschke, Pierre Denise, Gilles Clément

**Affiliations:** ^1^U.S. Navy Fellow, NASA Johnson Space Center, Houston, TX, United States; ^2^NASA Johnson Space Center, Houston, TX, United States; ^3^Brandeis University, Waltham, MA, United States; ^4^University of Caen Normandy, Caen, France; ^5^KBR, Houston, TX, United States

**Keywords:** sensorimotor system, bilateral vestibulopathy, parastronaut, spaceflight, disability, human systems integration

## Abstract

Upon landing after long-duration spaceflight, astronauts often experience motion sickness and impaired performance in mission-critical tasks such as egress, navigating obstacles, jumping, and recovering from falls. These changes are mainly attributable to central adaptations in their vestibular system. Current inflight countermeasures, which primarily focus on strength and endurance, are insufficient for preparing astronauts for postflight recovery. New countermeasures must be designed and tested to enable crewmembers to function without the extensive post-mission recovery support after landing on the Moon or Mars. Individuals with bilateral vestibulopathy are immune to motion sickness and might be better prepared for landing after spaceflight. They have adapted strategies for maintaining balance and orientation without relying on vestibular inputs, potentially making them more stable and less prone to disorientation in microgravity or rotating environments. Their unique adaptations may allow them to perform many mobility tasks more effectively during critical mission phases, such as vehicle egress, when other crew members might be more affected by vestibular issues. While they may not perform all tasks, these parastronauts can excel in specific roles that leverage their unique abilities, contributing to the mission’s success in specialized capacities. We propose using lunar gravity achieved during parabolic flight and prolonged centrifugation as models to study how functional task performance might be less impaired in parastronauts with bilateral vestibulopathy compared to healthy individuals when landing on the Moon after extended exposure to microgravity.

## 1 Introduction

As NASA expands its exploration missions to the Moon and Mars, there’s a growing emphasis on inclusivity and accessibility, particularly in adapting workspaces for people with disabilities. This evolution parallels the timeline of the Americans with Disabilities Act (ADA), passed in 1990, which mandated improved accessibility in workplaces and public spaces. The ADA aimed to ensure equal opportunities for people with disabilities to work and live without barriers. Today, individuals with disabilities are increasingly seen performing complex tasks alongside their more able-bodied peers (1).

The term ‘disability’ is defined herein as “long-term physical, mental, intellectual, or sensory impairments in interaction with various barriers” [(2), p. 1]. Disabilities may offer unique advantages, termed “hyperabilities,” in space environments. For example, individuals with visual impairments could potentially respond faster in emergencies occurring in darkness compared to their sighted counterparts. Integrating disabled individuals into otherwise able-bodied crews could enhance training and spacecraft design, such as creating accessible instrumentation beneficial for all crew members. People with disabilities also exhibit traits highly beneficial in space missions, including motivation, adaptability, resilience, and strong problem-solving skills. Moreover, employing astronauts with disabilities could enhance mission safety by providing valuable insights into managing contingencies during extended space missions, such as those planned for Mars exploration.

In 2021, SpaceX made history by sending the first person with a prosthesis into space. Recently, initiatives like AstroAccess have emerged to offer parabolic flights for individuals with disabilities. The term “parastronaut,” coined by the European Space Agency (ESA), defines an individual who meets the qualifications for and holds the official astronaut designation within a space agency’s astronaut corps. ESA introduced the term as part of their initiative to explore the feasibility of including astronauts with physical disabilities in future space missions (3, 4). In 2022, ESA selected John McFall, an above-the-knee amputee and orthopedic trauma surgeon, as the first parastronaut. McFall, who also competed in the Paralympic Games and won a bronze medal in sprinting in 2008, exemplifies the capabilities of individuals with disabilities (5, 6).

Exposure to altered gravity drives sensorimotor adaptation and learning for optimizing movement and spatial orientation in the novel environment. This adaptation is evident through symptoms such as motion sickness, spatial disorientation, decreased postural control and locomotion, and deficits in fine motor control following gravitational transitions (7). The vestibular organs play a crucial role in this adaptation, as evidenced by their significant role in terrestrial motion sickness and spatial disorientation. Inflight space motion sickness and re-entry motion sickness severity varies widely among crewmembers and can affect the completion of activities shortly after gravitational transitions (8, 9).

During spaceflight, space motion sickness (SMS) is typically associated with malaise, loss of appetite, headache, lack of initiative, impaired concentration, stomach awareness, and sudden vomiting without prodromal nausea (10). Bowel sounds are often decreased or absent in affected crewmembers (9). The incidence of SMS varies with spacecraft volume, ranging from few reported symptoms in the Mercury or Gemini programs to 73% in the Shuttle program (59, 60). Repeat flyers often experience decreased incidence and severity of SMS. Symptoms, which can be moderate to severe, generally last 2–3 days but may persist for more than a week (11), restricting critical activities like extra-vehicular activity during the initial mission days, and in some cases even longer.

**Figure 1:**
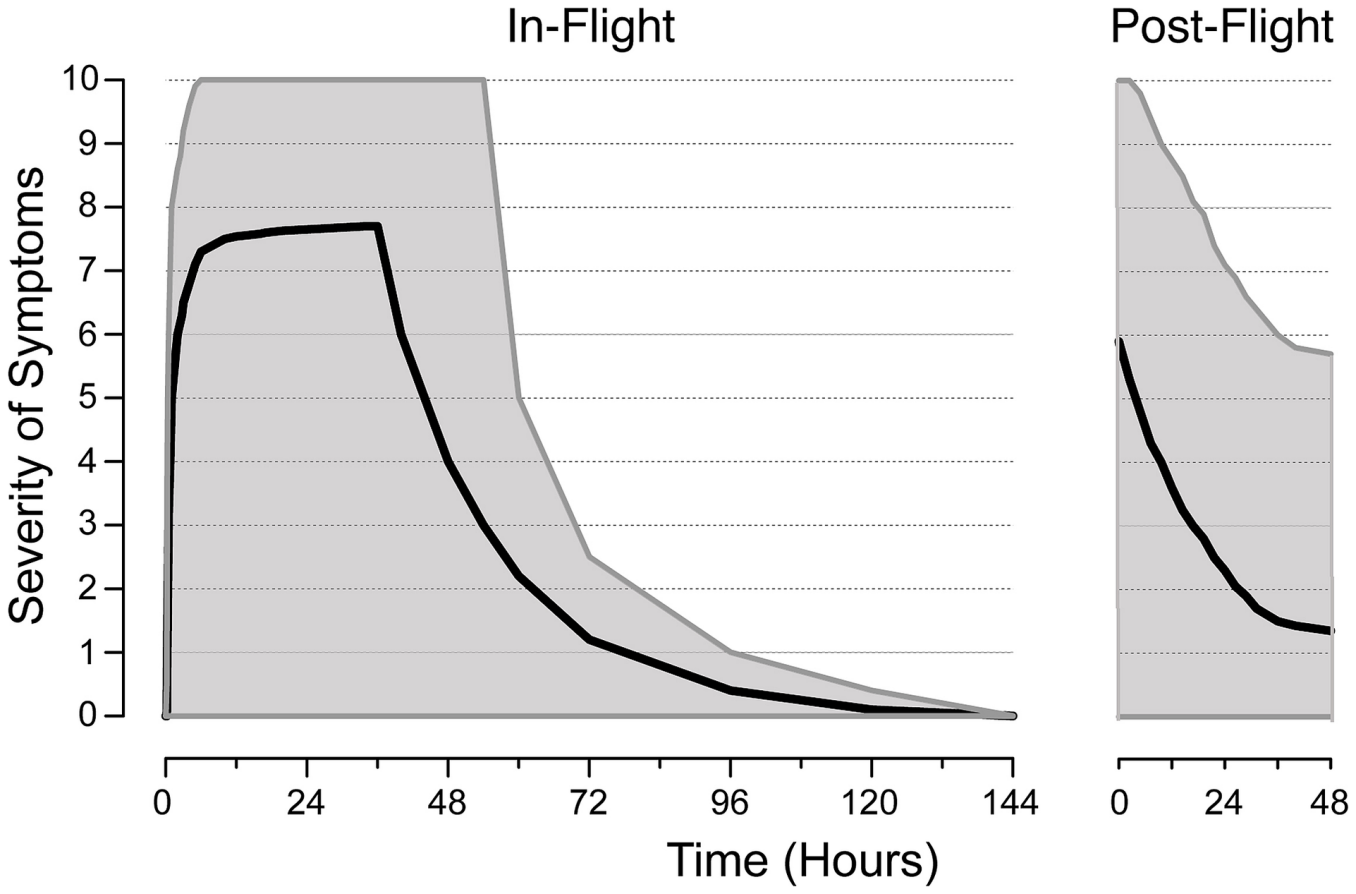
Typical severity of motion sickness during the early in-flight and post-flight periods of long-duration space missions. The severity of symptoms is measured on a scale from 0 (no symptoms) to 10 (vomiting). Medians (solid lines) and confidence intervals (gray areas) are shown. Note that flight rules restrict extravehicular activities before flight day three to allow for space motion sickness symptoms resolution. Adapted from Thornton et al. (9) and Clément and Wood (58).

Upon returning from spaceflight, approximately 15% of Space Shuttle astronauts experienced readaptation motion sickness, characterized by clumsiness, difficulty walking, and lingering after-effects (12). This incidence is higher and more severe following longer missions on board the International Space Station, with many astronauts experiencing nausea and vomiting. Both SMS and readaptation motion sickness have similar onsets, with symptoms appearing minutes after g-transition and persisting for several days, especially after long-duration flights (8). Provocative head movements and visual reorientation illusions can trigger in-flight vomiting (13). Factors like heat stress, dehydration, orthostasis, sleep deprivation, and exhaustion, as commonly observed post-flight, may increase susceptibility to re-entry motion sickness. However, unlike in-flight SMS, the incidence or severity of re-entry motion sickness does not consistently decrease with repeat flights, and a “relapse” phenomenon may occur post-flight (14) (Figure 1).

Bilateral vestibular loss has long been known to provide immunity to motion sickness (15). Parastronauts with bilateral vestibulopathy (BVP) would provide an opportunity to explore mechanisms of sensorimotor adaptation and potential countermeasures. This paper examines the benefits for parastronauts with BVP and identifies key areas in training and mission design that need to be addressed.

## 2 Disabilities to be considered for astronauts

### 2.1 Parastronaut feasibility foundational research study

In 2021, NASA commissioned the Potomac Institute for Policy Studies (PIPS) to investigate the feasibility of including parastronauts in space missions, focusing on individuals with lower leg deficiencies, short stature, or leg length differences. The study aimed to identify policy issues and outline necessary measures to ensure that qualified individuals with disabilities can participate in space missions safely and effectively. The study highlighted several considerations for parastronauts, such as modifying spacecraft seats and spacesuits for individuals with short stature, incorporating additional hand and footholds, and adapting adjustable prosthetics to manage fluid shifts in zero-gravity environments. It noted that parastronauts, especially those with lower leg prosthetics, may face limitations in extra-vehicular and surface activities due to increased energy expenditure and slower walking speeds compared to non-disabled individuals. The study recommended updating onboard training and emergency procedures to accommodate various types of disabilities. The study also recommended further research and testing to better understand and mitigate both human systems risks and engineering risks during such missions (16).

### 2.2 ESA study

In 2021, ESA committed to investing in and defining necessary adaptations of space hardware to enable highly qualified professionals with disabilities to participate safely in valuable space missions (17). The Parastronaut Feasibility Project (PFP) aimed to determine how to integrate parastronauts into human spaceflight without compromising mission objectives. Concurrently, ESA released a call for parastronauts, specifically targeting applicants under 50 years old with disabilities such as lower limb deficiencies, leg length differences, or short stature (<130 cm).

The ESA PFP study emphasized the need for meticulous hardware adaptation specific to each disability, indicating that design modifications must be individually reassessed and refined for each parastronaut candidate like John McFall, rather than applying a broad approach across disabilities or individuals. Key considerations highlighted by the PFP study included ingress and egress procedures, emergency response times, and spacecraft and spacesuit design modifications tailored to each disability (4). Some of the key lessons learned have focused on agreeing on realistic expectations for enabling a broad range of accessibility, adopting a flexible approach for training and human system design, and managing unconscious bias from the different stakeholders (3).

However, both the PIPS and ESA studies are limited in scope as they focus only on lower leg disabilities. Evaluating disabilities for space missions should not only aim for inclusivity but also enhance crew safety during unexpected in-mission events. Future spacecraft designs should consider a wider range of disabilities to make spaceflight more inclusive for parastronauts and improve the crew’s capability to handle medical emergencies in space. Heinicke et al. (18) recommended including disabilities such as upper limb deficiencies, upper leg deficiencies, paraplegia, multi-morbidity, blindness, deafness, and sensorimotor impairments in human spaceflight studies.

## 3 Studies with Gallaudet students: the deaf right stuff

Prior to the first human space flight, Dr. Ashton Graybiel (1902–1995) from the Naval Aviation Medical Research Laboratory (NAMRL) in Pensacola, Florida, began to test students with BVP from Gallaudet University on various motion stressors. The Ashton Graybiel Spatial Orientation Laboratory at Brandeis University in Waltham, Massachusetts, commemorates his contributions. Dr. Graybiel’s research focused on preventing motion sickness, which posed a threat to spaceflight, and he sought to understand the role of the vestibular organs in this syndrome by studying the differences between individuals with and without functional organs of equilibrium. Individuals with BVP lack functioning semi-circular canals and otolith organs, were particularly valuable for these studies.

Spatial disorientation and motion sickness are influenced by the visual, vestibular, and somatosensory systems. The vestibular system specifically detects acceleration, while vision and somatosensory information from skin, muscles, and joints provide supplementary cues. Eleven Gallaudet BVP subjects participated in a variety of tests, which included exposure to slow rotating rooms, a spatial disorientation device, elevator rides in the Empire State Building, parabolic flights, centrifuge spins, and journeys on ships in rough seas. The following provides an overview of the motion stressors and observations in individuals with BVP.

### 3.1 Rotating room

**Figure 2:**
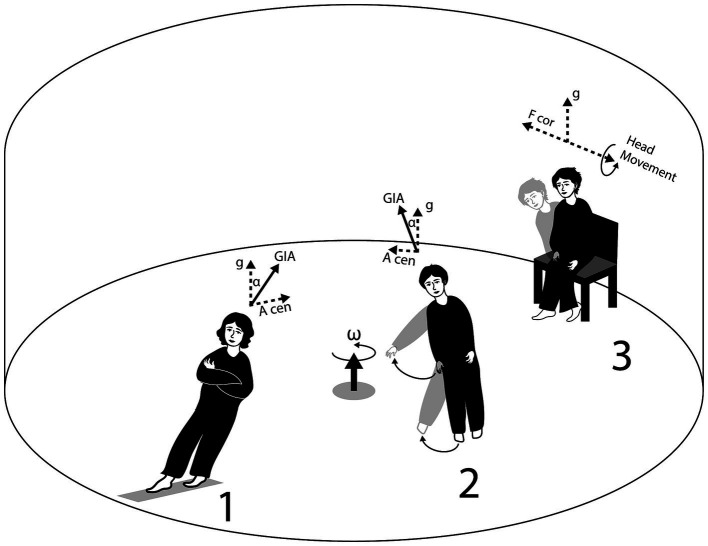
The Rotating Room facility of the Ashton Graybiel Spatial Orientation Laboratory, Brandeis University, demonstrates the accelerations experienced by subjects in different positions: standing (1), moving an arm or leg (2), or bending at the trunk (3). Key elements include g (acceleration due to gravity), A cen (centripetal acceleration), GIA (gravito-inertial acceleration, the resultant of g and A cen), F cor (Coriolis force), *ω* (angular velocity of Rotating Room), and *α* (the angle between gravito-inertial and gravitational upright). In position 3, the vectors representing g, GIA, and A cen are omitted to simplify the illustration. Credit: Olga Kuldavletova. Adapted from: https://brandeis.edu/graybiel/facilities/rotating-room/html.

The NAMRL Rotating Room facility, measuring 22 feet in diameter and 10 feet high, could rotate at speeds up to 20 rpm. Initially, at 12 rpm subjects seated in the room did not perceive centrifugal force. However, once they moved around, they had to maintain a ~ 30-degree angle from the vertical near the walls, facing challenges navigating across due to varying centrifugal forces Walking from one side to the other required a crouched posture, similar to ascending a steep hill. Objects tossed into the room would spin along the walls until reaching the bottom. After spending several days in motion, subjects underwent body-balancing tests upon returning to a stationary environment, including standing on one foot. Tests focused on their experiences during rotation, sensations before and after stopping, immediate reactions upon cessation, and subsequent Romberg and walking tests (Figure 2).

Most healthy individuals develop motion sickness symptoms when making head movements in a rotating room at rates exceeding 3 rpm and, through that experience, learn to limit these movements. Initially, their balance control is disrupted upon entering the slowly rotating environment, but it recovers within 3–4 days. After this period, most individuals can walk on thin rails, throw darts, and pour coffee without consciously thinking about motor control (19). When the slowly rotating room is stopped after a few days, healthy individuals experience after-effects and erroneous motion sensations during head movements. Their balance control is again disrupted for 3–4 days (20). The crucial role of the vestibular system in motion sickness was clearly demonstrated by the complete immunity to motion sickness and negligible change in performance observed in the Gallaudet students with BVP in the slow rotating room, contrasting with susceptibility in the healthy subjects (21, 22).

Interestingly, in these experiments, periodic stops of 10 to 15 min were required during long-duration slow rotating room runs for re-provisioning. Over time, the onboard healthy experimenters assisting with this activity were able to transition between the stationary and rotating environments without experiencing motion sickness or disruptions in movement control. They demonstrated perfect dual-adaptation, indicating that it is possible to simultaneously adapt to both rotating and non-rotating environments (23).

### 3.2 Coriolis acceleration platform

The Coriolis Acceleration Platform (CAP) was utilized to simulate the environment of a rotating space station. This circular room, situated on a 40-foot linear track, could rotate and move angularly, surpassing the capabilities of the Rotating Room. Its primary function was to investigate artificial gravity as a countermeasure to zero gravity experienced in space. The CAP lacked windows, preventing test subjects from visually perceiving the rotation. As a consequence, they felt stationary in a stationary environment. Objects thrown or rolled within the CAP demonstrated curved trajectories due to its rotational motion. Equipped with amenities like hot and cold running water, kitchen appliances, a toilet, television, bedding, and seating, the CAP provided a comfortable living environment. Engineers designed the CAP to simulate space station rotations at 10 rpm, while medical researchers studied its impact on physical and mental functions.

In August 1964, four individuals with BVP embarked on a more than two-week experiment inside the CAP. The study comprised three days of pre-testing, followed by 12 days of continuous spinning in one direction, with subsequent reversal direction spinning and a few days for assessment. Operational from 8:00 a.m. to 8:00 a.m., the CAP paused briefly each morning and evening for resupply and personnel changes. Lt. Robert Kennedy oversaw daily tests, having accumulated over 2,000 h aboard the CAP by August 1964. The BVP individuals, observed via footage, performed tasks with remarkable speed and accuracy despite the rotational environment. They rapidly adapted to walking within the spinning CAP. Throughout the experiment, the BVP individuals maintained their health and cognitive abilities, exhibiting no signs of physical deterioration. Daily tests consistently showed positive results, affirming their resilience to the rotational conditions.

Artificial gravity using centrifugation involves rotating a spacecraft or a section of it. This method can help reduce muscle atrophy, bone density loss, and other health issues related to long-duration spaceflight in microgravity. The centrifugal force generated by the rotation pushes objects, including astronauts, toward the outer edge of the rotating structure, simulating the effects of gravity. However, creating artificial gravity through centrifugation presents several challenges, such as the need for large rotating structures, managing the transition between rotating and non-rotating sections of the spacecraft, and addressing motion sickness and spatial disorientation that can affect movement and perception within the rotating habitat (24). Parastronauts with BVP are less affected by these issues and could therefore assist other crew members during their adaptation period to both rotating and non-rotating environments.

### 3.3 Human disorientation device

The Human Disorientation Device (HDD) was a seated cylindrical cab that could move simultaneously about a horizontal or vertical axis. Studies have compared the ability of healthy control subjects and individuals with BVP to set themselves to the postural vertical (25) and to set a line of light to the visual horizontal while tilted laterally ±90° from the vertical in 10° steps (26). Subjects, wearing eye patches to occlude vision, were passively tilted 30° to the left or right of the vertical. They then used a knob to actively control the chair motor and set themselves back to the vertical, completing a total of 30 trials. The performance of control subjects and those with BVP was not significantly different. Both groups showed their largest errors during the first 5 trials, but by trials 16–20, errors plateaued, with the groups’ results diverging by less than one degree.

Subjects were seated in a chair that was passively tilted to one of 19 possible positions, in random order, with angles ranging from 0° to ±90° in 10° increments. They were asked to align a line of lights, positioned approximately 36 inches in front of their heads, to the perceived visual horizontal using a knob. Both control and BVP subjects showed similar patterns in their responses as body tilt changed. However, BVP subjects generally exhibited larger errors at greater tilt angles compared to the control group. For the 19 tilt positions tested, the performance of the two groups did not significantly differ in more than half of the comparisons (25, 27).

The first study demonstrates that individuals with BVP, despite the absence of otolith function, determine the postural vertical with accuracy comparable to control subjects by relying on tactile, somatosensory, and proprioceptive cues. The second study, however, reveals that individuals with BVP show greater variability when setting a line of light to the horizontal in a darkened chamber. Together, one can infer from these studies that the absence of vestibular signals may not be critical for performance in a weightless environment.

The CAP was also used to study the oculogyral illusion, which occurs when an angular acceleration stimulates the semi-circular canals of the inner ear while the observer looks at a visual target. Under these conditions, a target that is stationary relative to the observer appears to shift in space. The target reaches a maximum displacement and then continues to seem in motion, but without further change in its position relative to the observer. Healthy individuals can experience this illusion at accelerations as low as 0.02 °/sec^2^, which is well below the threshold required to elicit nystagmus in the dark or for the observer to detect self-motion. Miller and Graybiel (28) found that individuals with BVP did not experience the oculogyral illusion, even at the highest acceleration tested (6 °/sec^2^). This suggests that parastronauts would not be susceptible to one of the most common visual illusions encountered in aviation and spaceflight.

### 3.4 Centrifuge runs

Exposure to altered gravitoinertial forces is common in both aviation and spaceflight, often resulting in visual illusions (such as the oculogravic illusion) and misperceptions of body orientation (like the somatogravic illusion). The severity of these illusions is directly related to the magnitude of the force change. These phenomena are among the most dangerous illusions encountered in both spaceflight and high-performance aviation (29). To better understand their effects, they have been systematically studied using centrifuges and slow-rotation rooms.

During exposure to a constant increase in gravitoinertial force, both visual and self-orientation illusions can occur. Notably, both individuals with normal vestibular function and those with BVP experience these illusions, although the latter group experiences them to a lesser extent. For both groups, there is a temporal delay before the somatogravic illusion reaches its full intensity, with similar onsets for both. However, the peak intensity of the illusion occurs after approximately 20 s for BVP subjects and about 2 min for those with intact vestibular function.

**Figure 3:**
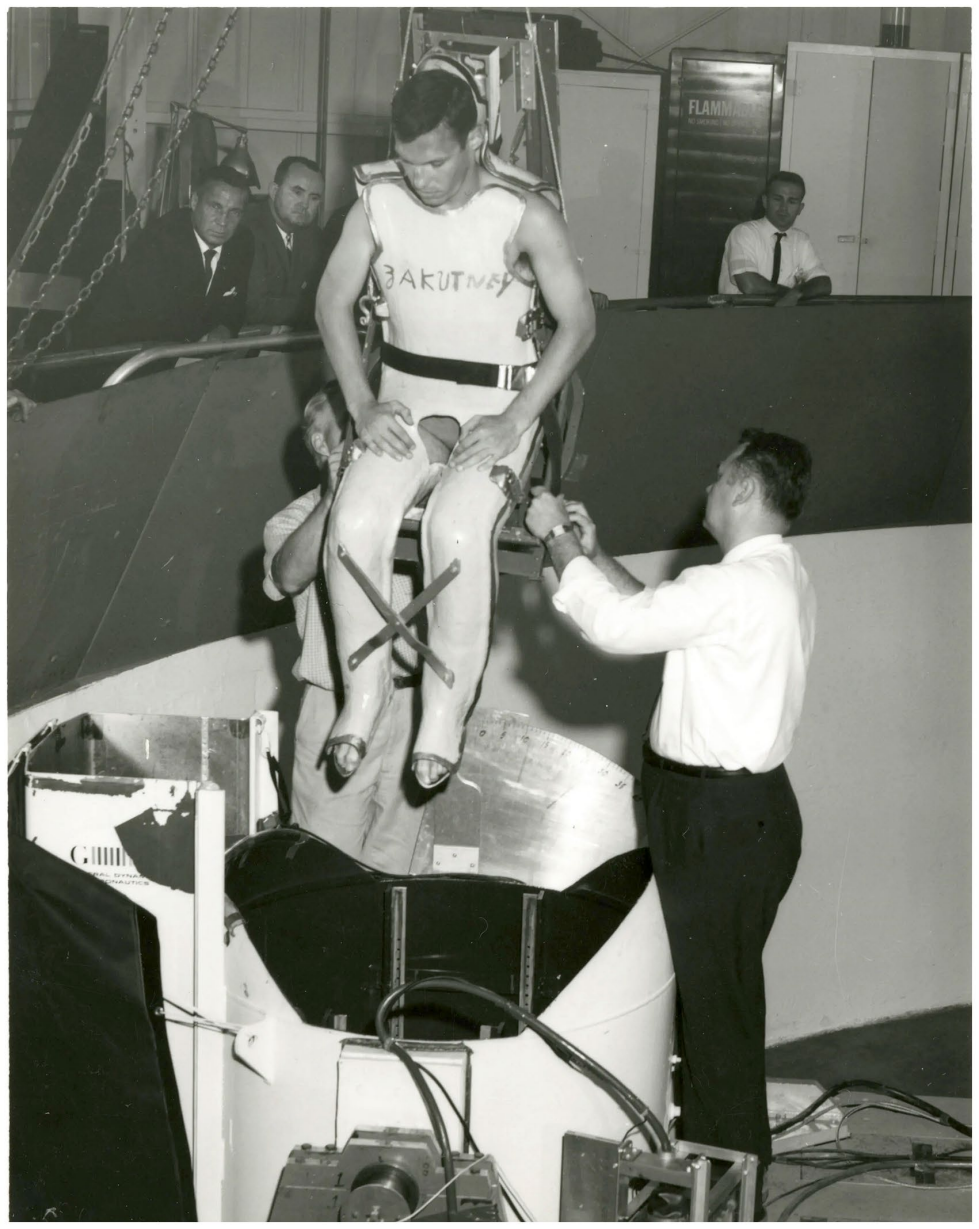
A vestibular patient in a fiberglass cast is being picked up by a crane and lowered into the water for a centrifuge run. Photo credit: NASA (https://www.nasa.gov/missions/project-mercury/how-11-deaf-men-helped-shape-nasas-human-spaceflight-program/).

Four Gallaudet students with BVP were tested in a gondola mounted at the end of a centrifuge at the General Dynamics Lab in San Diego. The subjects wore fiberglass suits designed to immobilize their bodies, allowing them to be securely bolted onto chairs. They were tested under both “dry” and “wet” conditions. In the wet conditions, the subjects were positioned the same way as in the dry conditions but were immersed up to their necks in a water tank (Figure 3). In one wet condition, they wore bathing trunks, and in the other, they wore wetsuits. The control subjects experienced large oculogravic illusions in all conditions, although the illusion was slightly less intense in the wet condition compared to the dry one. In contrast, the BVP subjects experienced much smaller oculogravic illusions than the control group in the dry conditions and no significant illusion at all in the wet conditions. Contact cues presumably played a key role in reducing the magnitude of oculogravic illusions for the BVP individuals (30). However, another factor may be an attenuation of signals from visceral and truncal graviceptors (31).

On Earth, when the head is laterally accelerated or tilted relative to gravity, it induces a torsional rotation of the eyes in the opposite direction of the acceleration or tilt. Over a range of 10–60° head tilts, the gain of this ocular counter-rolling is approximately 0.1. During centrifugation, ocular counter-rolling was observed in control subjects but not in individuals with BVP. Healthy subjects show decreased ocular counter-rolling in reduced gravity environments and increased counter-rolling in hypergravity (32), which can contribute to motion sickness and postural instability. However, the absence of ocular counter-rolling in parastronauts would not pose a significant issue in weightless conditions and could, in fact, be advantageous when exposed to variations in background force levels.

These findings suggest that parastronauts with reduced or absent vestibular function would be much less susceptible to the visual and postural illusions triggered by dynamic changes in gravitoinertial forces, compared to healthy individuals.

### 3.5 Parabolic flight

In 1962, Titov, during his participation in the second Soviet spaceflight, was the first to report experiencing a sensation of inversion while in a state of weightlessness. Subsequently, many astronauts and cosmonauts have reported feelings of inversion. During parabolic flight, subjects experience alternating periods of weightlessness and high gravitational forces, each lasting 20 to 30 s. Many seated passengers experience themselves as being inverted during the weightless period, especially during their first few parabolas. This inversion illusion can take different forms for different individuals. Some experience themselves as inverted and the aircraft as upright. Others feel and see both themselves and the aircraft as inverted. During spaceflights, astronauts may experience a shift in orientation as they alternate between looking at the spacecraft’s “floor” and gazing out at the Earth through a window (33).

Graybiel and Kellogg (34) compared the inversion illusion between healthy individuals and those with BVP. During the weightless phase of parabolic flight, subjects were either free-floating in the cabin or restrained in a fiberglass mold at various angles to the aircraft’s cabin (upright, 30°, 60°, and 90°). While control subjects experienced different forms of the inversion illusion, individuals with BVP consistently reported their orientation with respect to the cabin and the aircraft’s upright position with high accuracy.

The absence of the inversion illusion in parastronauts with BVP may offer an advantage, as it could decrease their susceptibility to disorientation caused by inversion illusions in weightless conditions or by shifting visual cues when moving between different compartments of the spacecraft.

### 3.6 Elevator rides

The BVP participants from Gallaudet University were exposed to varying levels of gravito-inertial forces in the high-speed elevators of the Empire State Building in New York City and the Waterman Building in Mobile, Alabama. In the Empire State Building, from 8:00 p.m. to 2:00 a.m., they took turns riding the express elevators between the first and 80th floors. During these rides, they experienced a 0.2 g reduction in gravity when descending and a 0.2 g increase above normal gravity when ascending. While riding, the subjects reported the onset and direction of motion under three different visual conditions: (a) viewing a small target light, (b) viewing an afterimage, and (c) viewing an afterimage superimposed on the target light.

The control subjects consistently reported the correct direction of motion. During downward acceleration of the elevators, the control subjects observed: (a) the target light moving downward, (b) the afterimage moving upward, and (c) a significant upward displacement of the afterimage, while the displacement of the real target was less clearly defined. Additionally, the control subjects’ eyes moved upward for about 150 milliseconds during downward acceleration. The opposite patterns of visual perception and eye movement were observed during upward acceleration of the elevators.

The BVP subjects did not experience visual displacements in any of the above conditions, nor did they exhibit reflexive eye movements in any of the conditions. However, they were consistently accurate in describing their direction of vertical motion (35). These findings indicate parastronauts with BVP would be less susceptible to the visual illusions evoked by gravitational force level transitions, and consequently less susceptible to spatial disorientation during the various phases of a spaceflight.

### 3.7 Journeys on ships in rough seas

Ten deaf test subjects were taken to the North Atlantic seas to study the residual effects of extreme motion and unusual stimulation. In 1964, they flew to North Sydney, Nova Scotia, and boarded the ferry Miquelon to the French Overseas Collectivity of Saint Pierre & Miquelon. After spending a few days in Saint Pierre, they returned on an overnight trip. The purpose was to determine if the vestibular-challenged subjects experienced symptoms of seasickness under the prevailing weather conditions. The tests included questionnaires, assessments of steadiness and ataxia before, during, and after the sea voyages, as well as collection of urine and blood for analysis. Despite rough seas and violent rocking of the ship, which prevented control subjects from taking some tests due to the severity of their symptoms even with the use of seasickness medication, the deaf test subjects did not experience seasickness.

## 4 Benefits for flying astronauts with bilateral Vestibulopathy

### 4.1 Selection criteria

In addition to the standard astronaut selection criteria, parastronaut candidates with BVP must meet specific conditions related to their diagnosis. Specific diagnostic criteria for bilateral vestibulopathy (BVP) have been provided by the Classification Committee of the Barany Society (36). The bilateral vestibular loss must be confirmed through various quantitative tests such as the caloric test, rotatory chair, ocular and cervical vestibular evoked myogenic potential tests (oVEMP, cVEMP), and video head impulse tests (vHIT), and it must symmetrically affect both the semicircular canal and otolith functions. The candidate should not experience any paroxysmal symptoms, including vertigo, nystagmus, tinnitus, or drop attacks. The condition must have been stable for at least two years. Furthermore, the vestibular loss should either be idiopathic or result from a non-progressive cause or disease that does not affect the hearing system or the central nervous system (e.g., CANVAS) (37).

### 4.2 Motion sickness

Motion sickness occurs when there is a mismatch between the sensory signals the brain receives about motion and orientation. On Earth, the vestibular system plays a crucial role in maintaining balance and spatial awareness by detecting motion and changes in gravity. In a microgravity environment, however, the normal gravitational cues that help with balance are absent. This disruption creates sensory conflicts between the visual, vestibular, and proprioceptive systems, leading to disorientation and an increased likelihood of symptoms such as dizziness, nausea, and vertigo, especially during activities involving head movements (13).

Over time, most astronauts’ brains adapt to the new sensory inputs in space, and the symptoms of space motion sickness typically subside as they undergo this adaptation process. However, upon re-entry, astronauts face difficulties in processing the sensory cues related to the high-g forces and the return to Earth’s gravity. Space and re-entry motion sickness pose a significant challenge, as they occur during crucial phases of a mission. To manage these symptoms, astronauts often rely on medications like antiemetics or take steps to minimize head and visual movements that could trigger discomfort.

In individuals with BVP, the complete loss of vestibular function reduces sensory conflicts, providing immunity to motion sickness in response to all forms of vestibular stimulation tested on Earth. Interestingly, even a partial loss of vestibular function—affecting just one side—significantly reduces susceptibility to motion sickness as well (38).

### 4.3 Emergency egress

Exposure to spaceflight produces adaptations in vestibular, sensorimotor, and cardiovascular systems that are maladaptive upon return to 1 g. These adaptations often manifest in balance and gait disturbances, cardiovascular deconditioning, and loss of muscle mass, muscle coordination and strength. Critical mission tasks after landing on a planetary surface may include a rapid egress from a vehicle. A combination of vestibular and sensorimotor alterations, reduced muscle strength, and presyncopal symptoms caused by orthostatic intolerance may inhibit timely execution of the egress (39).

While the countermeasures aboard the International Space Station (ISS), such as daily exercise, nutrition, and fluid management, are crucial for maintaining astronaut health, they are not fully efficient in completely countering the effects of microgravity (40). After today’s landing of the Soyuz and Crew Dragon capsules, a significantly large landing party assists the crew members during egress. The potential for re-entry motion sickness and spatial disorientation after a long-duration spaceflight is very high, making it difficult for crew members to exit the landing craft without help. A similar situation may occur after landing on the Moon, following prolonged adaptation to a microgravity environment. During the Apollo missions, astronauts did not report significant spatial disorientation or motion sickness on the lunar surface because the journey from Earth to the Moon took only a few days, and the crew did not have time to fully adapt to microgravity before landing. However, the current Artemis program plans for the crew to stay in orbit around the Moon for several weeks before landing, potentially increasing the risk of these issues.

Several spaceflight experiments have examined the performance of astronauts returning from long-duration ISS missions during the execution of an emergency egress, which requires tasks such as seat egress, walk and turn, open hatch, and jump. In particular, the time to complete seat egress, tandem walk, jump down, and recovery from a potential fall, were investigated in astronauts after short-duration Space Shuttle missions (41) and after long-duration ISS missions (42). The results from these experiments show clear alterations in the execution of these tasks after a two-week spaceflight on board the Space Shuttle, and after a six-month stay on board the ISS (43). In addition, significant impairments in the performance of these tasks have been observed in participants after a 70-day bed rest in the 6° head-down tilt position (44).

**Figure 4:**
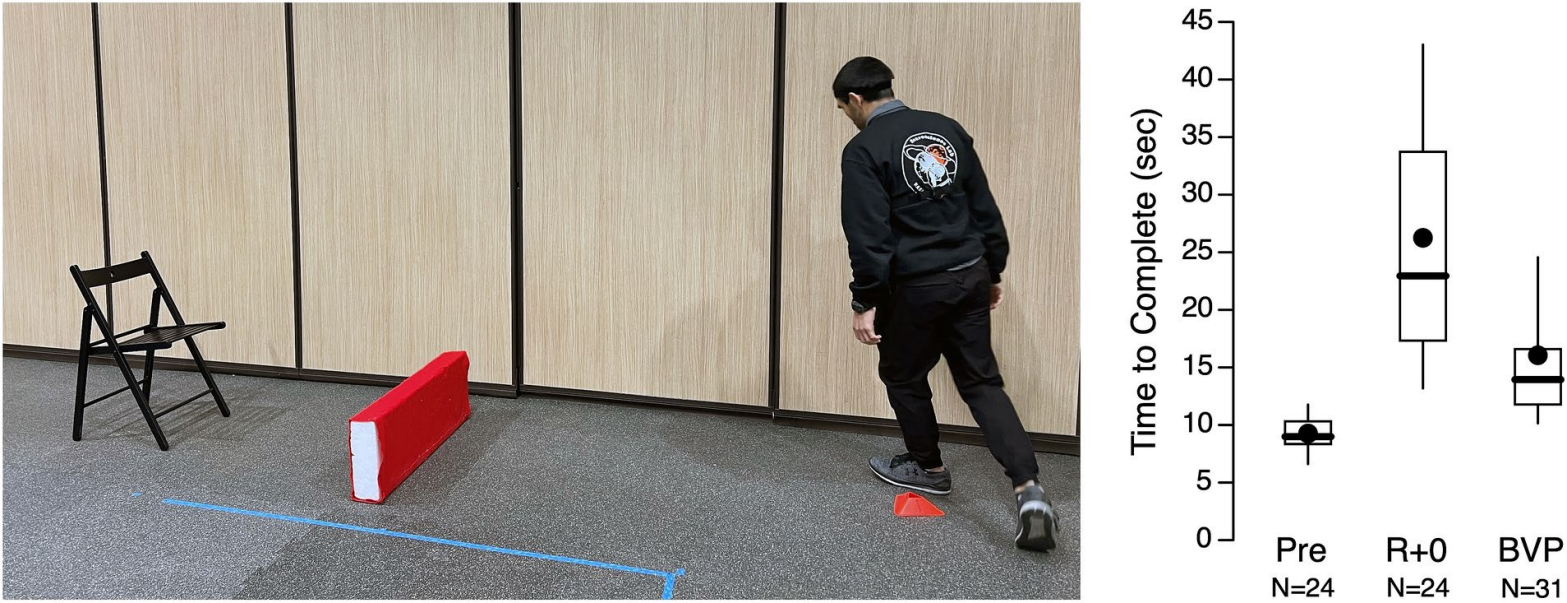
Time to complete a timed up and go test in astronauts before the flight (Pre), on the day following a long-duration spaceflight (R + 0), and in patients with bilateral vestibulopathy (BVP). The box and whisker plots display the interquartile range (box), the minimum and maximum values (whiskers), the median (thick horizontal line) and the mean (dot symbol) values. Adapted from Clément et al. (45).

In a ground-based study performed by our group in 2022, we compared the performance of functional tasks (Sit-to-Stand, Walk-and-Turn, Tandem Walk) between 30 subjects with BVP and 28 astronauts returning from 6–8 months stays aboard the International Space Station (45). The results showed that the BVP subjects took longer than healthy subjects to complete a modified the Walk-and-Turn test but completed it faster than astronauts on landing day (Figure 4). This result suggests that individuals with BVP may be more efficient when performing an emergency egress after adaptation to microgravity. However, the astronauts had recovered to levels comparable to BVP individuals one day after landing.

### 4.4 Other benefits

Individuals with bilateral vestibular loss often have associated hearing deficits. There have been instances where astronauts with hearing loss received waivers and successfully completed their missions. For instance, an astronaut who lost hearing in one ear due to a training accident, was granted a medical waiver by the chief flight surgeon to participate in an ISS mission (46). This case illustrates that astronauts with disabilities can still contribute effectively to space missions if they are otherwise qualified (16).

Moreover, designing space missions to accommodate individuals who are deaf could enhance crew readiness in handling trauma that may result in hearing loss. Implementing procedures and training that accommodate hearing impairments, such as learning sign language or utilizing redundant non-verbal communication methods, could better prepare crews for emergencies affecting hearing. Given the risk of decompression sickness or barotraumatic injuries during extra-vehicular activities, preparing for scenarios involving hearing impairment is essential (47).

Individuals with sensorimotor impairment, due to their experiences overcoming adversity and trauma, often exhibit personality traits that can greatly enhance the dynamics and effectiveness of a space crew (48). Their high level of resilience, developed through facing significant challenges, enables them to handle the demanding environment of space and stabilize the crew during tough situations. Additionally, their strong problem-solving skills, honed by navigating difficult circumstances, are invaluable for innovative thinking and finding solutions under pressure (23).

Adaptability is another key trait, as individuals with BVP had to adjust to new ways of living and functioning. This flexibility is beneficial in the challenging environment of space, where rapid adaptation is crucial. Their experiences often foster a deep sense of empathy, promoting team cohesion and effective communication. Traits such as determination, perseverance, and mental fortitude contribute to a culture of persistence and resilience. Furthermore, their diverse perspectives can enhance problem-solving and innovation within the crew. Integrating BVP parastronauts with these traits can enrich crew capabilities and underscore the value of diversity and inclusion in advancing human exploration (49).

Individuals who lose vestibular function gradually are able to adapt their movement control and balance despite the progressive loss. There are cases where individuals were unaware that they had lost function because they had been able to play baseball, tennis, ski, windsurf, without difficulty or clumsiness. Such individuals would be especially valuable in spaceflight and likely be able to adapt rapidly to low gravity and weightless conditions (50). However, the adaptability and resilience of parastronauts deserve deeper consideration for extended space missions.

While it is known that the total loss of vestibular junction confers immunity to motion sickness under all provocative forms of vestibular stimulation so far tested, it is also the case that half-sided loss greatly decreases susceptibility to motion sickness (38).

Dynamic visual acuity (DVA) is often impaired in individuals with vestibular dysfunction, as the vestibular system is essential for stabilizing vision during movement. Challenges in DVA can significantly impair critical tasks like navigation, emergency response, equipment monitoring, and spacewalks. A significant decline in DVA was observed in astronauts one day after returning from space, with a mean decrease of 0.75 eyechart lines (51). While this level of deterioration is comparable to what is commonly seen in patients with vestibular disorders, patients often develop strategies for dealing with this challenge (52).

## 5 Recommendations for ground-based studies

How would parastronauts with BVP be affected by spaceflight and lunar landing? We propose two series of experiments to evaluate the susceptibility of participants with BVP to motion sickness and their ability to egress a vehicle after a lunar landing.

### 5.1 Landing simulation

Motion sickness on Earth can be induced by prolonged exposure in a human centrifuge. Studies performed at TNO in the Netherlands exposed supine subjects (with feet pointing in the direction of motion) to acceleration in the fore and-aft direction of the subject’s body (X-direction) for up to 2 h at 3 g (53). During the centrifuge runs, subjects’ vitals were monitored, showing increased heart rate and blood pressure. After the centrifuge stopped, subjects experienced motion sickness symptoms and significant postural instability. They had difficulty taking corners or walking in a straight line, and with eyes closed, they tended to fall backward. Some subjects exhibited extreme visual dependency following centrifugation. These effects persisted for 6–12 h and resembled the symptoms of space and readaptation motion sickness (54).

The transition from 3 g to 1 g after centrifuge runs and from 1 g to 0 g during spaceflight both involve the body’s maladaptation to a “novel” gravitational environment. During prolonged centrifugation, the body eventually adapts to the hypergravity environment, becoming maladapted to the 1 g environment when exiting the centrifuge. This maladaptation leads to an inaccurate estimation of body state, resulting in motion sickness and inappropriate sensory-motor responses, such as postural over-corrections. A similar maladaptation occurs upon entry into weightlessness and upon return to Earth (53).

We propose to compare the responses of healthy subjects and those of individuals with BVP during and after a 1-h centrifuge run at 3 g. To simulate lunar gravity along their longitudinal body axis, subjects will be tilted head-up by 9.5 degrees (55). Throughout the runs, oxygen saturation of arterial blood, heart rate, and systolic and diastolic blood pressure will be continuously monitored and recorded. Immediately after the centrifuge stops, subjects will be instructed to stand for assessments of orthostatic intolerance, postural balance, and motion sickness severity. Following these tests, they will exit the centrifuge and perform critical mission tasks such as sit-to-stand, walk-and-turn, tandem walk with eyes open or closed, and jump from a 30 cm platform (42).

### 5.2 Parabolic flight in lunar gravity

Understanding how lunar gravity (0.16 g) affects the execution of functional tasks in crewmembers adapted to microgravity is critical for developing effective countermeasures and training astronauts. Parabolic flight is the only ground-based method that can create lunar gravity for sufficient durations to safely test changes in human perception and behavior. We propose to test individuals with BVP in lunar gravity during parabolic flights as a model for studying how the execution of functional tasks might be impaired in astronauts landing on the Moon after prolonged exposure to microgravity. These tasks would be the same as those previously used on astronauts returning from ISS missions and healthy subjects in partial gravity, including rising from a seated position, walking, jumping down, recovering from falls, and maintaining an upright stance (42).

**Figure 5:**
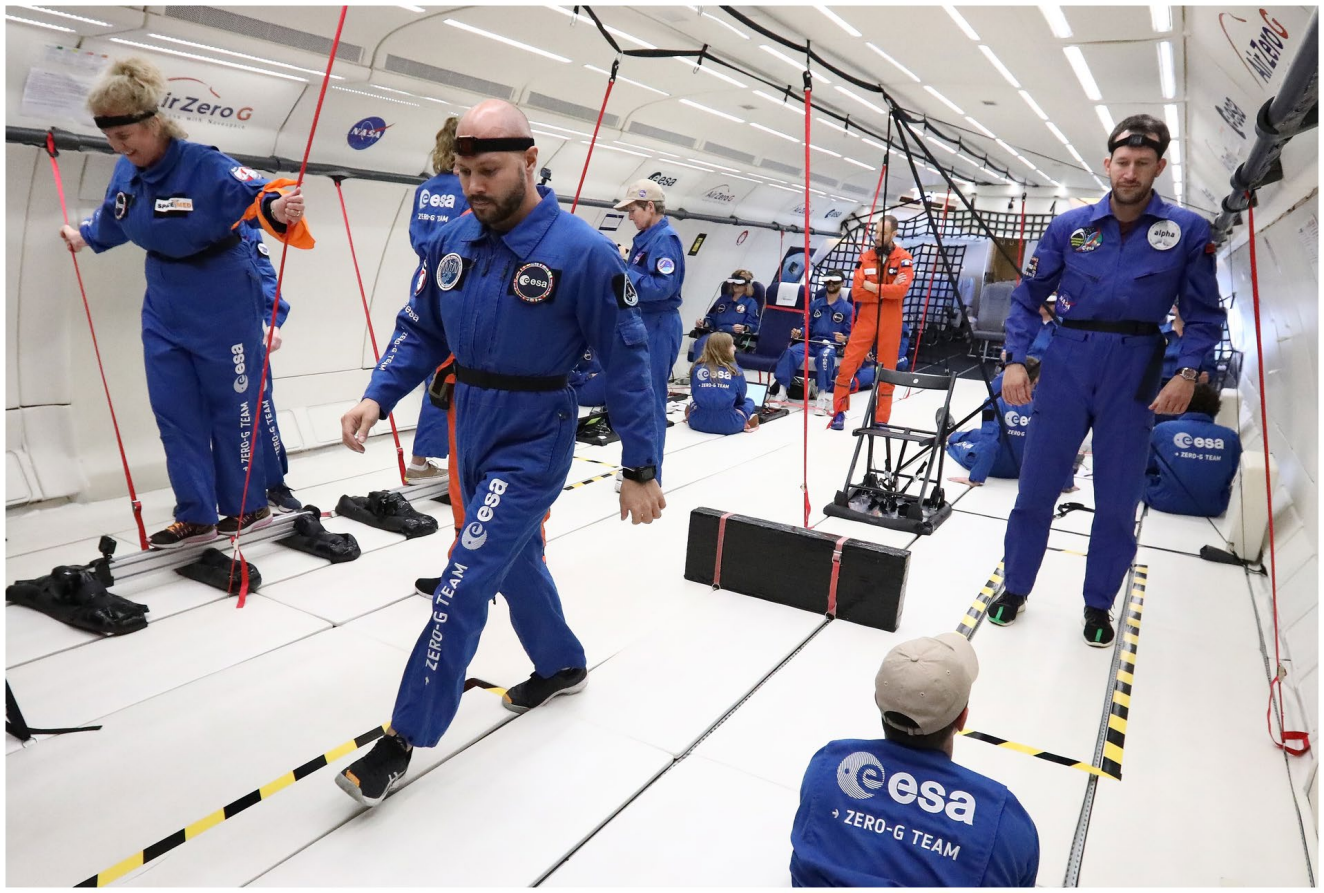
Healthy subjects executing functional tasks (left: Tandem Stance; center: Walk-and-Turn; right: Recovery-from-Fall) in partial gravity during the joint ESA-NASA campaign in June 2023. Consent was obtained from the individuals for the publication of this photo. Photo credit: Nicolas Montussi, NOVESPACE.

During a joint ESA-NASA parabolic flight campaign in June 2023, 12 healthy volunteers were tested in 10 parabolas that produced 0.25 g, 0.5 g, or 0.75 g aboard Novespace’s Zero-G aircraft, as well as at 1 g during level flight intervals between parabolas. The results indicated that reduced gravity altered the performance of activities such as settling after standing and navigating around obstacles (Figure 5). As gravity levels decreased, postural instability increased, resulting in longer times required to stand up, settle, walk, and negotiate obstacles (45). We hypothesize that BVP subjects would experience a smaller decrease in performance at lunar gravity compared to healthy subjects, as they rely less on proprioceptive inputs for their perception of upright. Consequently, parastronauts with BVP could assist other crew members in egressing the vehicle.

Studying how these BVP parastronauts adapt and perform after adaptation to microgravity and in lunar gravity can provide valuable insights into human physiology and adaptation, informing the development of better countermeasures and training programs for all astronauts.

## 6 Human systems integration

Human Systems Integration (HSI) provides comprehensive analysis, design, and assessment of requirements, concepts, and resources across seven domains. These domains include (1) the manpower needed and available for operation, maintenance, and training; (2) the cognitive and physical abilities required for personnel to perform their duties; (3) the training necessary to equip personnel with essential job skills and knowledge; (4) the incorporation of human characteristics into system design to optimize human-machine performance; (5) safety and occupational health measures to minimize errors and failures; (6) efforts to reduce system damage and personnel injury; and (7) the adaptation of the physical environment to meet personnel needs (56).

Human Factor Engineering (HFE) domain involves designing and evaluating system interfaces and operations for human well-being and optimized safety, performance and operability, while considering human performance characteristics as they affect and are affected by expected and unpredicted conditions (57).

The HSI and HFE domains provide the framework for successful integration of parastronauts to human spaceflight missions, including those with BVP. Designing for parastronauts can enhance redundancy and safety for all crew members by incorporating additional skills and resources. Early initiation of training for parastronauts is essential to account for unique operations, modifications to training materials, mission simulations, and team training. This preparation ensures both ground personnel and crewmates are ready for normal and unexpected events.

While some BVP parastronauts may be unable to perform some tasks such as extravehicular activities due to their disabilities, they may excel in other specialized roles and potentially use their disabilities as unique advantages. Mission planning might require specific pairings of parastronauts with other crew members, such as prioritizing crew members fluent in sign language to work with BVP parastronauts with hearing deficit.

Effective training involves having assigned mission crewmembers train and practice together to accommodate any necessary procedure modifications and build team confidence. Demonstrating the parastronaut’s ability to perform essential tasks during emergencies can alleviate any doubts or anxieties about flying with a parastronaut. Including both parastronauts and both HSI and HFE experts in the design and analysis processes can strategically optimize the research benefits of flying unique populations.

Incorporating parastronauts with BVP into space missions can enhance overall mission performance, safety, and understanding of human adaptation to space environments.
